# Understanding Ultrasound Power Doppler Synovitis at Clinically Quiescent Joints and Thermographic Joint Inflammation Assessment in Patients with Rheumatoid Arthritis

**DOI:** 10.3390/diagnostics14212384

**Published:** 2024-10-25

**Authors:** York Kiat Tan, Julian Thumboo

**Affiliations:** 1 Department of Rheumatology and Immunology, Singapore General Hospital, Outram Road, Singapore 169608, Singapore; 2 Duke-NUS Medical School, 8 College Rd, Singapore 169857, Singapore; 3 Yong Loo Lin School of Medicine, National University of Singapore, 10 Medical Dr, Singapore 117597, Singapore

**Keywords:** rheumatoid arthritis, ultrasonography, thermography, joints, disease activity, synovitis

## Abstract

**Background/Objectives**: Rheumatoid arthritis (RA) is a chronic autoimmune inflammatory arthritis. We aim to study subclinical PD synovitis and thermographic joint inflammation assessment in patients with RA. **Methods**: We compared (1) PD synovitis at clinically quiescent (non-swollen; non-tender) joints based on patients’ disease activity and (2) thermography (hands/wrists) outcomes based on PD joint inflammation findings and patient’s disease activity. **Results**: Among eighty RA patients (mean (SD) age 57.0 (12.6) years; 61 of whom (76.3%) were female), the wrists (62.7%), second metacarpophalangeal joints (MCPJs) (37.0%), third MCPJs (33.8%), fourth MCPJs (24.8%), and fifth MCPJs (20.9%) were the five joint sites most frequently displaying subclinical PD synovitis; with no statistically significance differences (*p* > 0.05) between patients with 28-joint disease activity score (DAS28) < 3.2 versus those with DAS28 ≥ 3.2. At these five joint sites bilaterally, (1) the total maximum (Total Tmax), total average (Total Tavg), and total minimum (Total Tmin) temperatures were significantly greater (*p* < 0.05) for Total PD (TPD) score >1 versus TPD score ≤ 1, while their area under the ROC curve (AUC) values in identifying TPD score >1 ranged from 0.789 to 0.810, and (2) Total Tmax, Total Tavg, Total Tmin, and TPD score were significantly greater (*p* < 0.05) for patients with DAS28 ≥ 3.2 versus those with DAS28 < 3.2. **Conclusions**: Our results would serve as useful background data in studies on RA monitoring strategies detecting subclinical PD synovitis. Thermographic temperatures were greater in patients with greater disease activity and can help discriminate ultrasound PD joint inflammation severity.

## 1. Introduction

Rheumatoid arthritis (RA) is a chronic autoimmune inflammatory arthritis affecting around 0.5% to 1% of the population worldwide and is more commonly encountered in women than men (ratio 3:1) [1,2,3]. Early diagnosis and institution of appropriate treatment (e.g., disease-modifying anti-rheumatic drugs (DMARDs) therapy) are key to improving the treatment outcomes for patients with RA [4]. In the last two decades, there has been much knowledge gained in the application of ultrasound and magnetic resonance imaging (MRI) as diagnostic, prognostic, and disease-monitoring tools in patients with rheumatoid arthritis (RA) [5]. The advantage of these modern imaging tools is that they can directly visualize the synovial inflammation and bone erosion at the joints of patients with RA [6]. Both imaging tools have been shown to be superior in the detection of joint/tendon inflammation when compared to clinical examination of the joints in patients with RA [7]. About a decade ago, the European League Against Rheumatism (EULAR) provided a set of recommendations for the use of imaging in the clinical management of patients with RA, which spans the use of ultrasound and MRI as diagnostic, prognostic, and outcome measurement tools [8]. For example, EULAR has recommended that both ultrasound and MRI can be used to detect inflammation that predicts subsequent joint damage, even in clinical remission, and can be used to assess persistent inflammation [8,9,10,11].

Ultrasound PD synovitis is a poor prognostic marker, and treating this has been shown to reduce flares in RA patients in clinical remission/low disease activity [9,10,12]. An unmet clinical need in RA patient care, therefore, is the present lack of established consensus or guidelines on how often to assess for ultrasound PD joint inflammation and which joints should be included in these assessments among RA patients who have attained clinical remission or low disease activity.

Although ultrasound (and MRI) are well-established imaging modalities for joint inflammation assessment in patients with RA [13,14,15], they are not without their limitations [16,17]. Sonographers typically need a substantial period of training before gaining proficiency in musculoskeletal ultrasonography and scanning multiple different joint sites using ultrasound can be time-consuming. MRI, on the other hand, has magnet-related contraindications (e.g., pacemaker use) and its cost makes it less feasible as a routine monitoring tool in patients with RA [16,17,18]. Hence, there is a need to look at other affordable low-cost imaging modalities that may be feasible for use in the routine rheumatology practice setting [19]. Thermography, an emerging imaging modality, helps assess joint inflammation in RA by objectively quantifying joint surface temperatures [20]. Thermal imaging is contactless, non-invasive, and allows rapid image acquisition, with modern thermal cameras being compact, portable, and straightforward to use [19,21].

In this study, to help better characterize subclinical joint inflammation, we aimed to determine in which clinically quiescent (non-swollen and non-tender) joints are ultrasound PD synovitis most frequently encountered in patients with RA and whether these may differ based on patients’ disease activity. An additional aim was to evaluate the role of thermography in comparison with ultrasonography for the assessment of joint inflammation as well as the patient’s disease activity.

## 2. Materials and Methods

This is a single-center, cross-sectional, and observational study conducted at a tertiary referral hospital. Patients with RA included in this study (1) were either male or female patients aged 21 to 99 years old, (2) fulfilled the 2010 EULAR/American College of Rheumatology (ACR) RA classification criteria [22], (3) had disease duration less than 2 years and (4) were on first-line conventional disease-modifying anti-rheumatic drugs (DMARDs). Pregnant patient(s) were excluded from the study. Eligible patients were consecutively recruited at the hospital outpatient rheumatology clinic between December 2020 and November 2023. This study received approval by our local Centralised Institutional Review Board (CIRB) and adheres to the relevant research ethical guidelines. All patients gave their informed consent before enrolment into the study.

### 2.1. Clinical Assessment

The clinical and imaging (thermal and ultrasound) assessments were performed during the same study visit. Clinical joint examinations, including the 28-joint disease activity score (DAS28) assessment, were performed by trained rheumatology nurses (after receiving standardized training) who were blinded to the results of thermal and ultrasound imaging. Joint tenderness and swelling were graded as either absent or present. The patients were categorized into the following two RA patient groups: (1) those with clinical remission/low disease activity (DAS28 < 3.2) and (2) those with at least moderate disease activity (DAS28 ≥ 3.2). We chose the cut-off score of 3.2 for DAS28 since DAS28 less than 3.2 is used clinically to define low disease activity/clinical remission in patients with RA [9,10].

### 2.2. Imaging Assessment

**Figure 1:**
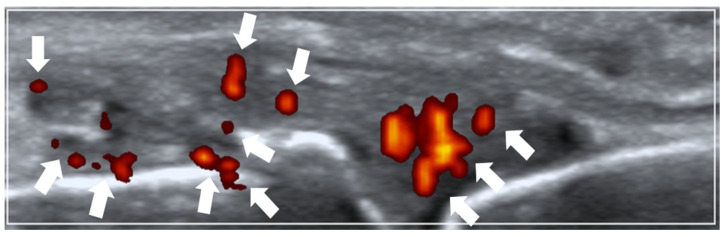
Ultrasound power Doppler (PD) joint inflammation. Example of a sonogram (longitudinal view) showing grade 2 PD joint inflammation at the metacarpophalangeal joint (arrows pointing towards PD vascularity).

A 26-joint ultrasonography was performed based on the EULAR guidelines [23] at the following joint sites bilaterally: (1) dorsal recesses of all metacarpophalangeal joints (MCPJs), (2) dorsal recesses of the thumb’s interphalangeal joint and the remaining fingers’ proximal interphalangeal joints, (3) dorsal recesses of the wrist’s (i) distal radio-ulnar and (ii) radio-carpal/inter-carpal joints, (4) humero-radial and posterior fossa recesses of the elbow joint, and (5) supra-patellar recess of the knee joint. A single rheumatologist experienced in musculoskeletal ultrasonography carried out the ultrasound imaging. Thermal imaging was performed by a separate trained study team personnel while being blinded to the ultrasound findings. The Mindray M9 (Mindray Bio-Medical Electronics Co., Ltd., Shenzhen, China) ultrasound machine (with an L14-6Ns linear probe) was utilized with the following ultrasound scan settings: pulse repetition frequency (PRF) of 700 Hz and Doppler frequency of 5.7 MHz. Ultrasound PD joint inflammation severity was scored semi-quantitatively (0–3) according to the validated EULAR–Outcome Measures in Rheumatology (EULAR–OMERACT) ultrasound scoring method [24,25] (see Figure 1 for an example of scoring ultrasound PD joint inflammation).

**Figure 2:**
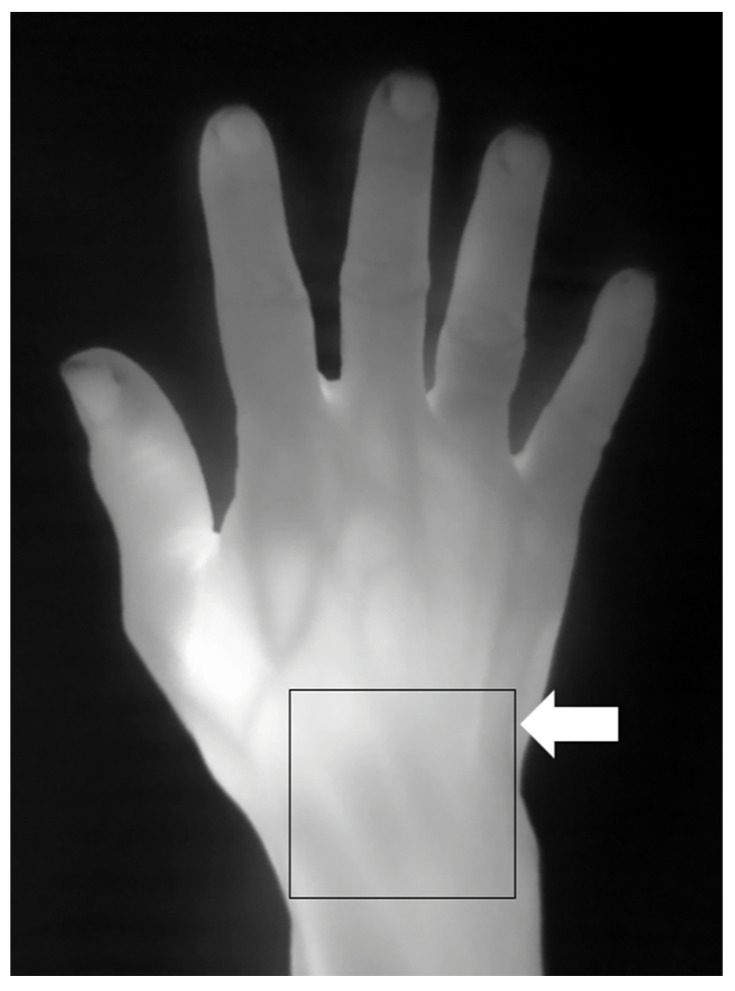
Thermography showing a right-hand thermogram. Example of thermal imaging assessment (arrow showing region of interest (ROI) manual segmentation) at the dorsal aspect of the wrist.

Thermography followed established imaging methods previously described in the literature [19,26,27]. Standardized thermal imaging was conducted in a draft-free room (without windows) with an ambient temperature of around 23 °C [26]. A high-performance portable thermal camera FLIR T640 (FLIR Systems AB, Sweden) was utilized for thermal imaging with the following thermal camera settings: predefined emissivity value of 0.98 for skin [19]; thermal sensitivity of <30 milli-Kelvin (mK) at 30 °C; pixel resolution 640 × 480). As per usual practice, before thermal imaging was carried out, patients were rested for 15 min to allow for acclimatization [26]. Physical objects (such as jewelry and watches) obscuring the view of the thermal camera were removed. The patient’s hands (neutral position) were placed on a flat tabletop. The dorsal view of the hand was imaged with the thermal camera placed 50 cm above the hand. Using a region of interest (ROI) [19,27,28] manual segmentation approach (see Figure 2 for an example of ROI manual segmentation for thermography), rectangular boxes representing the ROIs were placed over the target joint sites (e.g., the wrist, MCPJ, etc.) and the corresponding thermographic temperatures (maximum (Tmax), average (Tavg), minimum (Tmin) temperatures) at the ROIs recorded for further analysis.

### 2.3. Intra-Observer Reliability Analysis for Thermography

Unlike ultrasound, which is a well-validated [24,25] imaging modality for joint inflammation assessment in RA, there has been much less reliable data with regard to thermographic assessment of joint inflammation in RA. Hence, we carried out intra-observer reliability testing (single observer) for thermal imaging at joint sites most commonly harboring subclinical PD synovitis (e.g., wrists and MCPJs). A random sample of baseline thermograms including 32 manually segmented ROIs (consisting of 16 wrist ROIs and 16 MCPJ ROIs) were retrieved for manual resegmentation (at least 2 weeks from the baseline manual segmentation). The thermographic temperatures Tmax, Tavg, and Tmin were used for intra-observer reliability testing.

### 2.4. Statistical Analysis

A joint is considered to have subclinical PD synovitis if it is clinically quiescent (non-swollen; non-tender) and shows PD positivity [29,30]. At joint sites commonly harboring subclinical PD synovitis, (1) the frequency of joint(s) with subclinical PD synovitis were compared between patients with DAS28 < 3.2 versus those with DAS28 ≥ 3.2 using the Pearson’s chi-square test and (2) the summed thermographic temperatures (total maximum (Total Tmax), total average (Total Tavg), and total minimum (Total Tmin) temperatures) were compared between total PD (TPD) score (sum of PD sub-scores) > 1 versus TPD score ≤ 1 using the 2-independent sample t-test; while the ability of Total Tmax, Total Tavg, and Total Tmin to identify TPD score > 1 was evaluated using receiver operating characteristic (ROC) analysis. For the ROC analysis, the ‘Closest to Top Left’ method was applied to help determine the optimal ROC curve cut-off value. The threshold value of >1 for the TPD score was chosen due to the observation that patients with degenerative joint disease such as hand osteoarthritis may also display low-level (grade 1) ultrasound PD signal [31]. Additionally, the summed thermographic temperatures (Total Tmax, Total Tavg, and Total Tmin) and the TPD score were compared between patients with DAS28 ≥ 3.2 versus those with DAS28 < 3.2 using the 2-independent sample *t*-test. The intra-class correlation coefficient (ICC) was utilized to measure the intra-observer reliability (single observer) for the following thermographic parameters: Tmax, Tavg, and Tmin. ICC results interpretation were as follows: <0.40 (low); between 0.40 and 0.75 (moderate); 0.75 to 0.90 (substantial); >0.90 (excellent) [32]. Statistical significance was set at *p* < 0.05. All statistical analyses were performed using IBM SPSS Statistics for Windows, version 26 (IBM Corp., Armonk, NY, USA).

## 3. Results

### 3.1. Patients’ Baseline Characteristics

A total of 2080 joints from 80 RA patients were examined. The patients’ baseline characteristics are as follows: mean (SD) age of patients was 57.0 (12.6) years; 61 patients were female (76.3%); 61 patients were Chinese (76.3%); 43 patients were rheumatoid factor (RF) positive (53.8%); 44 patients were anti-cyclic citrullinated peptide (anti-CCP) positive (55.0%); mean (SD) disease duration of the patients was 6.2 (5.8) months; mean (SD) DAS28 of the patients was 3.69 (1.36); 15 patients had DAS28 < 2.6 (18.8%), 23 patients had DAS28 2.6 to <3.2 (28.8%), and 42 patients had DAS28 ≥ 3.2 (52.5%); 53 patients (66.3%) were on oral prednisolone; all patients were on one or more of the following disease-modifying anti-rheumatic drug (DMARDs): methotrexate, leflunomide, sulfasalazine, and/or hydroxychloroquine.

### 3.2. Frequency of Joint Sites with PD Synovitis at Non-Swollen and Non-Tender Joints

**Figure 3:**
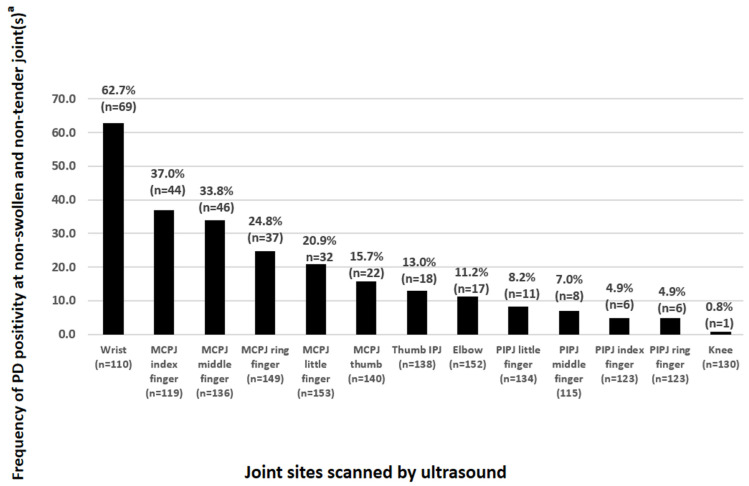
Relative frequency of joint sites with PD synovitis at non-swollen and non-tender joints. Abbreviations: PD, power Doppler; MCPJ, metacarpophalangeal joint; IPJ, interphalangeal joint; PIPJ, proximal interphalangeal joint. ^a^ Ranked in descending order of frequency.

Figure 3 summarizes the frequency (in percentages) of PD-positive joint(s) at clinically quiescent (non-swollen and non-tender) joints assessed by 26-joint ultrasonography. The 5 joint sites most frequently displaying ultrasound PD positivity at non-swollen and non-tender joints were (in descending order) the wrists (62.7%), second metacarpophalangeal joints (MCPJs) (37.0%), third MCPJs (33.8%), fourth MCPJs (24.8%), and fifth MCPJs (20.9%); with no statistically significance differences (all *p*-values > 0.05) between the two DAS28 patient groups (see Table 1). Among larger joint sites (Figure 3), 11.2% and 0.8% of the clinically quiescent (non-swollen and non-tender) elbow and knee joints showed ultrasound PD positivity, respectively.

### 3.3. Comparison Between Thermal Imaging and Ultrasound Imaging

At the bilateral 5 joint sites most frequently harboring subclinical PD synovitis (see Table 2), the Total Tmax, Total Tavg, and Total Tmin were all significantly greater (*p* < 0.05) for TPD score > 1 versus TPD score ≤ 1 (Total Tmax: mean (SD) TPD score > 1 versus TPD score ≤ 1 were 321.3 (22.1) and 298.8 (20.0), respectively, *p* = 0.007; Total Tavg: mean (SD) TPD score > 1 versus TPD score ≤ 1 were 310.7 (22.1) and 290.0 (18.3), respectively, *p* = 0.013; Total Tmin: mean (SD) TPD score > 1 versus TPD score ≤ 1 were 301.9 (22.7) and 282.0 (17.2), respectively, *p* = 0.019).

### 3.4. Comparing Imaging Outcomes Between Patients from the Two DAS28 Categories

At the bilateral 5 joint sites most frequently harboring subclinical PD synovitis (see Table 3), the Total Tmax, Total Tavg, Total Tmin, and TPD score were all significantly greater (*p* < 0.05) for DAS28 ≥ 3.2 versus DAS28 < 3.2 (Total Tmax: mean (SD) DAS28 ≥ 3.2 versus DAS28 < 3.2 were 324.9 (19.2) and 312.6 (24.9), respectively, *p* = 0.015; Total Tavg: mean (SD) DAS28 ≥ 3.2 versus DAS28 < 3.2 were 314.21 (19,2) and 302.4 (24.4)), respectively, *p* = 0.019; Total Tmin: mean (SD) DAS28 ≥ 3.2 versus DAS28 < 3.2 were 305.4 (19.9) and 293.9 (24.8)), respectively, *p* = 0.024; TPD score: DAS28 ≥ 3.2 versus DAS28 < 3.2 were 7.7 (5.5) and 5.2 (4.4), respectively, *p* = 0.028).

### 3.5. ROC Curve Analysis

**Figure 4:**
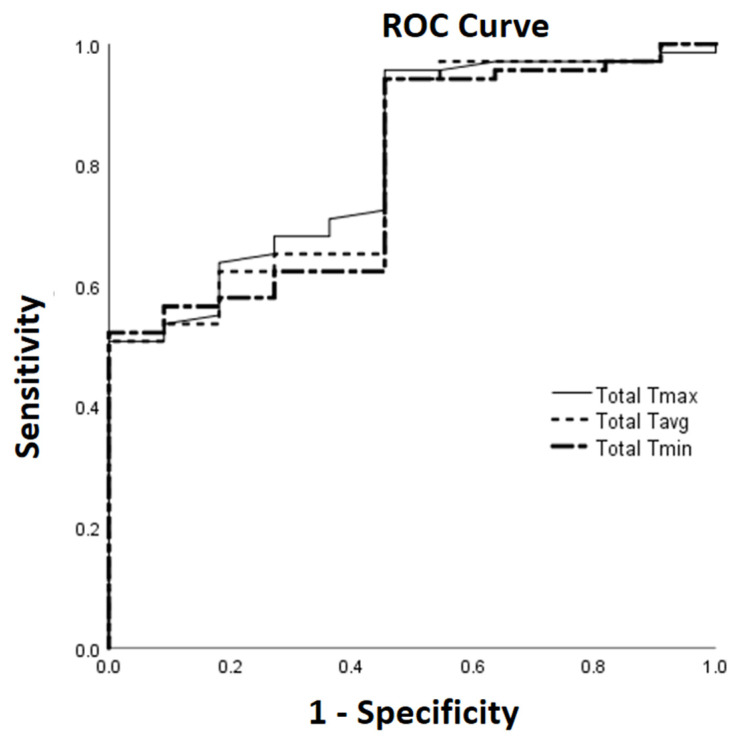
Use of thermographic parameters to identify TPD score > 1 applying ROC analysis. Abbreviations: ROC, receiver operator characteristic; Tmax, maximum temperature; Tavg, average temperature; Tmin, minimum temperature.

The ROC curves for Total Tmax, Total Tavg, and Total Tmin in identifying TPD score > 1 are shown in Figure 4. The area under the ROC curves (AUCs) and their 95% CI using optimal cut-off levels of ≥285.3, ≥279.2, and ≥271.5 in identifying TPD score > 1 were 0.810 (0.687, 0.933), 0.800 (0.671, 0.928), and 0.789 (0.660, 0.918) for Total Tmax, Total Tavg, and Total Tmin, respectively. The corresponding sensitivity (Sn), specificity (Sp), positive predictive value (PPV), and negative predictive value (NPV) using the above optimal cut-off levels in identifying TPD score > 1 for the thermographic temperatures are as follows: Total Tmax, Sn 95.7%, Sp 54.5%, PPV 93.0%, and NPV 66.7%; Total Tavg, Sn 94.2%, Sp 54.5%, PPV 92.9%, and NPV 60%; Total Tmin, Sn 94.2%, Sp 54.5%, PPV 92.9%, and NPV 60%.

### 3.6. Intra-Observer Reliability Testing

The ICC results (see Table 4) from the subset of wrist and MCPJ ROIs were high for all the thermographic parameters (Tmax, Tavg and Tmin) and ranged from 0.995 to 0.998 (for Tmax: ICC 0.997, 95% CI 0.994 to 0.998); for Tavg: ICC 0.998, 95% CI 0.995 to 0.999); for Tmin: ICC 0.995, 95% CI 0.991 to 0.998).

## 4. Discussion

In our study, we have identified the five joint sites most frequently displaying PD positivity at clinically quiescent (non-swollen and non-tender) joints (ranging from 20.9% to 62.7%) to be the wrist, second MCPJ, third MCPJ, fourth MCPJ, and fifth MCPJ. Interestingly, there are no significant differences in the frequency of PD positivity among non-swollen and non-tender joints at these five joint sites comparing RA patients in clinical remission or low disease activity (DAS28 < 3.2) versus those with at least moderate disease activity (DAS28 ≥ 3.2). Our data suggests that joints harboring subclinical PD synovitis commonly occur in RA patients regardless of a higher or lower disease activity state. While one might argue that joints harboring subclinical PD synovitis among those with a higher state of disease activity (e.g., DAS28 ≥ 3.2) might not truly matter (since a higher disease activity in itself is already an indication for treatment escalation), the converse may not be true in patients with a lower state of disease activity (e.g., DAS28 < 3.2). In the 2022 update of the EULAR recommendations for DMARDs use in RA [33], clinical remission or low disease activity remains to be the treatment goal in patients with RA. At present, though we have the imaging capability to detect joints harboring subclinical synovitis (such as through the use of ultrasound) and know that RA patients in clinical remission or low disease activity harboring ultrasound PD synovitis can have worse disease outcomes (e.g., disease flares and bone damage progression) [9,10], there has been no established clinical algorithm(s) nor clear guideline(s) on how best we can monitor RA patients in states of clinical remission or low disease activity for the presence of ultrasound PD synovitis. Our results on joints displaying subclinical PD synovitis would therefore serve as useful background data in studies on RA monitoring strategies employing ultrasonography in the detection of joints with subclinical PD synovitis. For example, the wrist joints are far and away the commonest site affected by subclinical PD synovitis, so it appears that the wrists should be included in any set of joints to be scanned for subclinical PD inflammation assessment. Moreover, it appears that scanning the hands alone detects the majority of subclinical PD synovitis. An important question that arises from our study is whether thermography could be a good substitute for ultrasound in this context (i.e., detection of subclinical PD synovitis) as it would be much easier to implement in practice. At present, ultrasound is an established imaging modality for the assessment of joint inflammation in patients with RA [34,35,36], while thermography is an emerging imaging tool still requiring validation in the assessment of joint inflammation in patients with RA [19].

In this study, we have also compared thermography with ultrasonography for joint inflammation assessment. To the best of our knowledge, our study is the first to demonstrate that thermography can help discriminate ultrasound PD joint inflammation severity at joint sites most commonly harboring subclinical PD synovitis. Specifically, at the bilateral five joint sites most frequently harboring subclinical PD synovitis, the summed thermographic temperatures were all significantly higher with greater ultrasound PD joint inflammation severity (i.e., comparing TPD score > 1 versus TPD score ≤ 1); while the AUCs from the ROC analysis in identifying a greater ultrasound PD joint inflammation severity (i.e., TPD score > 1) ranged from 0.789 to 0.810. Additionally, we have shown that at the bilateral five joint sites most frequently harboring subclinical PD synovitis, the summed thermographic temperatures and TPD score were all significantly higher in patients with DAS28 ≥ 3.2 versus those with DAS28 < 3.2. For thermography, studies have shown that RA patients have different thermographic temperature profiles when compared to healthy controls [19,37,38], although there has been less data on the use of thermal imaging in discriminating joint inflammation severity in patients with RA [19,21]. A recent RA hand/wrist study by Morales-Ivorra et al. [21] applying a machine learning-based automated technique to assess joint inflammation using hand thermograms obtained an AUC of 0.78 with 95% CI of 0.71 to 0.86 (*p* < 0.001) in identifying active synovitis detected on ultrasound. Taken together, the study by Morales-Ivorra I et al. [21] and our study suggest that thermography is a promising tool that can help discriminate ultrasound-detected joint inflammation severity at the hand/wrist joints in patients with RA, although this needs to be further evaluated in other RA cohorts. Unlike our study, where patients followed a period of acclimatization prior to thermal imaging, the study by Morales-Ivorra et al. [21] had no acclimatization process to simulate real-world conditions. Further well-designed studies will be necessary to clarify the importance of having an acclimatization protocol for thermographic assessment of joint inflammation in patients with RA.

Our study is not without its limitations. In this monocentric study, both thermography and ultrasonography were performed at a single time point applying a cross-sectional study design. Moreover, we have included only RA patients with early disease (less than 2 years duration) and who are on conventional DMARDs. RA patients with early disease (less than 2 years duration) were chosen in our study as current RA guidelines [4,33] emphasize the importance of detecting RA patients early so that appropriate treatment may be instituted promptly. Moreover, choosing RA patients with early disease will allow a more homogenous patient population pool to be evaluated in our present study. Future RA studies with a prospective longitudinal study design with clinical and imaging assessments performed at multiple time points for comparative analysis will be required, and ideally tested out in RA patients with different clinical profiles such as those with longstanding disease and those on biological DMARDs. Our study demonstrated high intra-observer consistency (single observer) for the measurement of thermographic temperatures (Tmax, Tavg, and Tmin) as evidenced by the excellent ICC results. Future studies with two or more observers will be required to obtain data on inter-observer reliability for thermographic temperature measurement in patients with RA. Another limitation of the present study is the lack of a control group for comparison. Future RA imaging studies should ideally include an appropriate control group (which could be either healthy volunteers or patients with osteoarthritis) for comparison.

## 5. Conclusions

In summary, we have added to the RA literature by describing the frequency of ultrasound PD positivity at clinically quiescent (non-swollen and non-tender) joints at various joint sites evaluated through the use of a 26-joint ultrasonography. At the five joint sites most frequently displaying subclinical PD synovitis, (1) there appears to be no significant differences in the frequency of PD positivity among clinically quiescent (non-swollen and non-tender) joints comparing RA patients in the two DAS28 patient groups and (2) thermography of the hand/wrist was able to help discriminate ultrasound PD joint inflammation severity. Additionally, the summed thermographic temperatures and TPD score were all significantly higher in patients with DAS28 ≥ 3.2 versus those with DAS28 < 3.2. Our results on joints displaying subclinical PD synovitis would serve as useful background data in studies on RA monitoring strategies employing ultrasonography in the detection of subclinical synovitis. Thermography appears promising for joint inflammation assessment at the hand/wrist of patients in RA and will require further validation in other independent RA cohorts.

## Figures and Tables

**Table 1:** Comparing PD-positive non-swollen and non-tender joints according to DAS28 categories.

Joint Site ^a^		DAS28 < 3.2 (Frequency ^b^ of PD Positivity Among Non-Swollen and Non-Tender Joints in %)	DAS28 ≥ 3.2 (Frequency ^b^ of PD Positivity Among Non-Swollen and Non-Tender Joints in %)	*p*-Value ^c^
Wrist	Right	*n* = 16 out of 26 (61.5%)	*n* = 18 out of 27 (66.7%)	0.697
	Left	*n* = 20 out of 31 (64.5%)	*n* = 15 out of 26 (57.7%)	0.598
MCPJ index finger	Right	*n* = 11 out of 30 (36.7%)	*n* = 10 out of 26 (38.5%)	0.890
	Left	*n* = 14 out of 33 (42.4%)	*n* = 9 out of 30 (30.0%)	0.306
MCPJ middle finger	Right	*n* = 10 out of 31 (32.3%)	*n* = 14 out of 35 (40.0%)	0.514
	Left	*n* = 8 out of 33 (24.2%)	*n* = 14 out of 37 (37.8%)	0.221
MCPJ ring finger	Right	*n* = 7 out of 34 (20.6%)	*n* = 11 out of 41 (26.8%)	0.529
	Left	*n* = 7 out of 34 (20.6%)	*n* = 12 out of 40 (30.0%)	0.356
MCPJ little finger	Right	*n* = 4 out of 35 (11.4%)	*n* = 9 out of 40 (22.5%)	0.206
	Left	*n* = 8 out of 35 (22.9%)	*n* = 11 out of 43 (25.6%)	0.780

Abbreviations: DAS28, 28-joint disease activity score; PD, power Doppler; MCPJ, metacarpophalangeal joint. ^a^ The 5 joint sites most frequently harboring subclinical PD synovitis were selected for comparative analysis. ^b^ Only PD positivity at non-swollen, non-tender joints were included in the calculation. ^c^ Comparative analysis performed using Pearson’s chi-square test.

**Table 2:** Comparative analysis of thermographic parameters for the two PD score categories.

Thermographic Parameter	Mean (SD) Within Ultrasound Category		Difference (95% CI)	*p*-Value ^a^
	TPD Score > 1	TPD Score ≤ 1		
Total Tmax	321.3 (22.1)	298.8 (20.0)	22.5 (6.3–38.8)	0.007 **
Total Tavg	310.7 (22.1)	290.0 (18.3)	20.7 (4.6–36.9)	0.013 *
Total Tmin	301.9 (22.7)	282.0 (17.2)	19.9 (3.4–36.5)	0.019 *

Abbreviations: Tmax, maximum temperature; Tavg, average temperature; Tmin, minimum temperature; TPD, total power Doppler. ^a^ Comparative analysis performed using the 2-independent samples *t*-test. Statistically significant: * *p* < 0.05, ** *p* < 0.01.

**Table 3:** Comparative analysis of imaging parameters for the two DAS28 patient categories.

Imaging Parameter	Mean (SD) Within DAS28 Category		Difference (95% CI)	*p*-Value ^a^
	DAS28 ≥ 3.2	DAS28 < 3.2		
Total Tmax	324.9 (19.2)	312.6 (24.9)	12.3 (2.5, 22.1)	0.015 *
Total Tavg	314.2 (19.2)	302.4 (24.4)	11.7 (2.0, 21.5)	0.019 *
Total Tmin	305.4 (19.9)	293.9 (24.8)	11.5 (1.6, 21.5)	0.024 *
TPD score	7.7 (5.5)	5.2 (4.4)	2.5 (0.3, 4.8)	0.028 *

Abbreviations: Tmax, maximum temperature; Tavg, average temperature; Tmin, minimum temperature; TPD, total power Doppler. ^a^ Comparative analysis performed using the 2-independent samples *t*-test. Statistically significant: * *p* < 0.05.

**Table 4:** Intra-observer reliability testing for thermography.

Thermographic Parameter	Intra-Class Correlation Coefficient	95% CI
Tmax	0.997	0.994 to 0.998
Tavg	0.998	0.995 to 0.999
Tmin	0.995	0.991 to 0.998

Abbreviations: Tmax, maximum temperature; Tavg, average temperature; Tmin, minimum temperature.

## Data Availability

All new data were published in this article.

## References

[1] Smolen J.S., Aletaha D., McInnes I.B. (2016). Rheumatoid arthritis. Lancet.

[2] Huang J., Fu X., Chen X., Li Z., Huang Y., Liang C. (2021). Promising Therapeutic Targets for Treatment of Rheumatoid Arthritis. Front. Immunol..

[3] Favalli E.G., Biggioggero M., Crotti C., Becciolini A., Raimondo M.G., Meroni P.L. (2019). Sex and Management of Rheumatoid Arthritis. Clin. Rev. Allergy Immunol..

[4] Fraenkel L., Bathon J.M., England B.R., St Clair E.W., Arayssi T., Carandang K., Deane K.D., Genovese M., Huston K.K., Kerr G. (2021). 2021 American College of Rheumatology Guideline for the Treatment of Rheumatoid Arthritis. Arthritis Rheumatol..

[5] Ranganath V.K., Hammer H.B., McQueen F.M. (2020). Contemporary imaging of rheumatoid arthritis: Clinical role of ultrasound and MRI. Best Pract. Res. Clin. Rheumatol..

[6] Tan Y.K., Ostergaard M., Bird P., Conaghan P.G. (2014). Ultrasound versus high field magnetic resonance imaging in rheumatoid arthritis. Clin. Exp. Rheumatol..

[7] Baker J.F., Conaghan P.G., Gandjbakhch F. (2018). Update on magnetic resonance imaging and ultrasound in rheumatoid arthritis. Clin. Exp. Rheumatol..

[8] Colebatch A.N., Edwards C.J., Ostergaard M., van der Heijde D., Balint P.V., D’Agostino M.A., Forslind K., Grassi W., Haavardsholm E.A., Haugeberg G. (2013). EULAR recommendations for the use of imaging of the joints in the clinical management of rheumatoid arthritis. Ann. Rheum. Dis..

[9] Han J., Geng Y., Deng X., Zhang Z. (2016). Subclinical Synovitis Assessed by Ultrasound Predicts Flare and Progressive Bone Erosion in Rheumatoid Arthritis Patients with Clinical Remission: A Systematic Review and Metaanalysis. J. Rheumatol..

[10] Foltz V., Gandjbakhch F., Etchepare F., Rosenberg C., Tanguy M.L., Rozenberg S., Bourgeois P., Fautrel B. (2012). Power Doppler ultrasound, but not low-field magnetic resonance imaging, predicts relapse and radiographic disease progression in rheumatoid arthritis patients with low levels of disease activity. Arthritis Rheum..

[11] Tan Y.K., Chew L.C., Allen J.C., Lye W.K., Htay L.L., Hassan A., Conaghan P.G., Thumboo J. (2018). Utility of ultrasonography in guiding modification of disease modifying anti-rheumatic drugs and steroid therapy for inflammatory arthritis in routine clinical practice. Int. J. Rheum. Dis..

[12] Zhao J., Wang Y., Geng Y., Zhang X., Deng X., Ji L., Song Z., Zhang Z. (2020). Intensive therapy alleviates subclinical synovitis on ultrasound and disease activity and reduces flare in rheumatoid arthritis patients who have achieved clinical target—A randomized controlled trial. Semin. Arthritis Rheum..

[13] Di Matteo A., Mankia K., Azukizawa M., Wakefield R.J. (2020). The Role of Musculoskeletal Ultrasound in the Rheumatoid Arthritis Continuum. Curr. Rheumatol. Rep..

[14] McQueen F.M., Chan E. (2014). Insights into rheumatoid arthritis from use of MRI. Curr. Rheumatol. Rep..

[15] Tan Y.K., Li H., Allen J.C., Thumboo J. (2020). Anti-cyclic citrullinated peptide but not rheumatoid factor is associated with ultrasound-detected bone erosion among rheumatoid arthritis patients with at least moderate disease activity. Int. J. Rheum. Dis..

[16] Rowbotham E.L., Grainger A.J. (2011). Rheumatoid arthritis: Ultrasound versus MRI. AJR Am. J. Roentgenol..

[17] Rubin D.A. (2019). MRI and ultrasound of the hands and wrists in rheumatoid arthritis. I. Imaging findings. Skelet. Radiol..

[18] Tan Y.K., Conaghan P.G. (2012). Insights into osteoarthritis from MRI. Int. J. Rheum. Dis..

[19] Kow J., Tan Y.K. (2023). An update on thermal imaging in rheumatoid arthritis. Jt. Bone Spine.

[20] Ahalya R.K., Snekhalatha U., Dhanraj V. (2023). Automated segmentation and classification of hand thermal images in rheumatoid arthritis using machine learning algorithms: A comparison with quantum machine learning technique. J. Therm. Biol..

[21] Morales-Ivorra I., Narváez J., Gómez-Vaquero C., Moragues C., Nolla J.M., Narváez J.A., Marín-López M.A. (2022). Assessment of inflammation in patients with rheumatoid arthritis using thermography and machine learning: A fast and automated technique. RMD Open.

[22] Aletaha D., Neogi T., Silman A.J., Funovits J., Felson D.T., Bingham C.O., Birnbaum N.S., Burmester G.R., Bykerk V.P., Cohen M.D. (2010). 2010 rheumatoid arthritis classification criteria: An American College of Rheumatology/European League Against Rheumatism collaborative initiative. Ann. Rheum. Dis..

[23] Backhaus M., Burmester G.R., Gerber T., Grassi W., Machold K.P., Swen W.A., Wakefield R.J., Manger B., Working Group for Musculoskeletal Ultrasound in the EULAR Standing Committee on International Clinical Studies including Therapeutic Trials (2001). Guidelines for musculoskeletal ultrasound in rheumatology. Ann. Rheum. Dis..

[24] D’Agostino M.A., Terslev L., Aegerter P., Backhaus M., Balint P., Bruyn G.A., Filippucci E., Grassi W., Iagnocco A., Jousse-Joulin S. (2017). Scoring ultrasound synovitis in rheumatoid arthritis: A EULAR-OMERACT ultrasound taskforce-Part 1: Definition and development of a standardised; consensus-based scoring system. RMD Open.

[25] Terslev L., Naredo E., Aegerter P., Wakefield R.J., Backhaus M., Balint P., Bruyn G.A.W., Iagnocco A., Jousse-Joulin S., Schmidt W.A. (2017). Scoring ultrasound synovitis in rheumatoid arthritis: A EULAR-OMERACT ultrasound taskforce-Part 2: Reliability and application to multiple joints of a standardised consensus-based scoring system. RMD Open.

[26] Chojnowski M. (2017). Infrared thermal imaging in connective tissue diseases. Reumatologia.

[27] Lerkvaleekul B., Jaovisidha S., Sungkarat W., Chitrapazt N., Fuangfa P., Ruangchaijatuporn T., Vilaiyuk S. (2017). The comparisons between thermography and ultrasonography with physical examination for wrist joint assessment in juvenile idiopathic arthritis. Physiol. Meas..

[28] Nosrati Z., Bergamo M., Rodríguez-Rodríguez C., Saatchi K., Häfeli U.O. (2020). Refinement and validation of infrared thermal imaging (IRT): A non-invasive technique to measure disease activity in a mouse model of rheumatoid arthritis. Arthritis Res. Ther..

[29] Ogishima H., Tsuboi H., Umeda N., Horikoshi M., Kondo Y., Sugihara M., Suzuki T., Matsumoto I., Sumida T. (2014). Analysis of subclinical synovitis detected by ultrasonography and low-field magnetic resonance imaging in patients with rheumatoid arthritis. Mod. Rheumatol..

[30] Huang Y., Liu K.J., Chen G.W., Liu J.F., Mo F.Q., Xie Y.H. (2022). Diagnostic value of semi-quantitative grading of musculoskeletal ultrasound in wrist and hand lesions of subclinical synovitis in rheumatoid arthritis. Am. J. Nucl. Med. Mol. Imaging.

[31] Kortekaas M.C., Kwok W.Y., Reijnierse M., Stijnen T., Kloppenburg M. (2016). Brief Report: Association of Inflammation with Development of Erosions in Patients with Hand Osteoarthritis: A Prospective Ultrasonography Study. Arthritis Rheumatol..

[32] Dibai-Filho A.V., Oliveira A.K., Oliveira M.P., Barros M.A., Bevilaqua-Grossi D., Guirro R.R.J. (2022). Reliability of quantitative sensory testing on myofascial trigger points in the upper trapezius muscle of individuals with chronic neck pain. Rev. Assoc. Med. Bras..

[33] Smolen J.S., Landewé R.B.M., Bergstra S.A., Kerschbaumer A., Sepriano A., Aletaha D., Caporali R., Edwards C.J., Hyrich K.L., Pope J.E. (2023). EULAR recommendations for the management of rheumatoid arthritis with synthetic and biological disease-modifying antirheumatic drugs: 2022 update. Ann. Rheum. Dis..

[34] Koppikar S., Diaz P., Kaeley G.S., Eder L. (2023). Seeing is believing: Smart use of musculoskeletal ultrasound in rheumatology practice. Best Pract. Res. Clin. Rheumatol..

[35] Grainger A.J., Rowbotham E.L. (2013). Rheumatoid arthritis. Semin. Musculoskelet. Radiol..

[36] Ishizaki J. (2024). Assessment of Musculoskeletal Ultrasound of Rheumatoid Arthritis. Methods Mol. Biol..

[37] Gatt A., Mercieca C., Borg A., Grech A., Camilleri L., Gatt C., Chockalingam N., Formosa C. (2020). Thermal characteristics of rheumatoid feet in remission: Baseline data. PLoS ONE.

[38] Gatt A., Mercieca C., Borg A., Grech A., Camilleri L., Gatt C., Chockalingam N., Formosa C. (2019). A comparison of thermographic characteristics of the hands and wrists of rheumatoid arthritis patients and healthy controls. Sci. Rep..

